# Computed Tomography-Guided Radiofrequency Ablation of Nasal Carcinomas in Dogs

**DOI:** 10.3390/ani14243682

**Published:** 2024-12-20

**Authors:** María Dolores Alférez, Andrea Corda, Ignacio de Blas, Lucas Gago, Telmo Fernandes, Ignacio Rodríguez-Piza, Beatriz Balañá, Plamena Pentcheva, Javier Caruncho, Alicia Barbero-Fernández, Jorge Llinás, David Rivas, Amaia Escudero, Pablo Gómez-Ochoa

**Affiliations:** 1 VetCorner Unavets, 50012 Zaragoza, Spain; lolaalferez77@gmail.com (M.D.A.); pablogomezochoa@gmail.com (P.G.-O.); 2 Department of Veterinary Medicine, University of Sassari, 07100 Sassari, Italy; plamenapentcheva@gmail.com; 3 Department of Animal Pathology, University of Zaragoza, 50013 Zaragoza, Spain; deblas@unizar.es; 4 Department of Mathematics and Computer Science, University of Barcelona, 08007 Barcelona, Spain; lgagogag69@alumnes.ub.edu; 5 Imaginologia Veterinaria do Porto, 4490-479 Porto, Portugal; insidevetecografia@gmail.com; 6 Anicura Glòries Hospital Veterinari, 08010 Barcelona, Spain; ignasi.rodriguez@anicura.es; 7 Hospital Anicura Aralar Veterinarios, 50410 Zaragoza, Spain; bboncovet@gmail.com (B.B.); david.rivas@anicura.es (D.R.); amaia.escudero@anicura.es (A.E.); 8 Arealonga Veterinary Clinic, 15190 A Coruña, Spain; javicaruncho@gmail.com; 9 Department of Diagnostic Imaging, European University, 28670 Madrid, Spain; aliciabarbero.vet@gmail.com; 10 Hospital Anicura Valencia Sur, 46460 Valencia, Spain; jorgellinasformacion@gmail.com

**Keywords:** nasal carcinoma, diagnostic imaging, cancer therapy, thermoablation, radiofrequency ablation, canine, minimally invasive therapy, veterinary oncology, CT-guided procedure

## Abstract

Nasal carcinomas in dogs are locally invasive neoplasms with a low metastatic rate that pose significant treatment challenges due to their location and aggressiveness. This study evaluates the safety, feasibility, and therapeutic outcomes of computed tomography-guided radiofrequency ablation (CT-guided RFA) in 15 dogs diagnosed with nasal adenocarcinoma. All patients underwent staging and histopathological diagnosis before treatment. CT-guided RFA achieved a significant tumor volume reduction (82.8%) and improvement in clinical signs such as nasal discharge, epistaxis, and respiratory distress, without complications. Post-RFA CT examinations demonstrated a significant decrease in Hounsfield units and tumor volume. This study has shown that CT-guided RFA is an effective cytoreductive option for minimally invasive management of nasal adenocarcinomas in dogs, particularly when traditional therapies like radiation therapy or surgery are not feasible.

## Simple Summary

Nasal carcinomas in dogs are aggressive tumors that are challenging to treat due to their location. This study investigates the use of radiofrequency ablation (RFA), a minimally invasive technique, to manage these tumors. Fifteen dogs were treated, and the results showed a significant reduction in tumor size and improvement in clinical signs like nasal discharge and breathing difficulties. The procedure was safe, with no major complications, and follow-up imaging confirmed its effectiveness. RFA can be considered a viable alternative treatment for nasal carcinoma in dogs when radiation therapy or surgery is not possible.

## 1. Introduction

Canine nasal tumors are characterized by their locally aggressive nature and relatively low metastatic rate, estimated to be between 10% and 40%, depending on the type of neoplasm and disease progression [1,2,3,4]. Although the incidence of primary intranasal tumors in dogs is low, accounting for approximately 1–2% of all canine neoplasms, they present a significant challenge in veterinary oncology due to their location, invasiveness, and high recurrence rate, which exceeds 60% in most cases [5,6,7,8]. Carcinomas are the most prevalent type of intranasal tumors, comprising about two-thirds of all cases, with adenocarcinomas and squamous cell carcinomas being the most frequently observed [2,6,9]. These tumors are predominantly seen in middle-aged to older dogs, particularly in dolichocephalic and mesocephalic breeds, and are more common in males [6,10]. Despite the low metastatic rate at diagnosis, metastases to regional lymph nodes and lungs are often present at the time of death in 40–50% of cases [1,3,10]. Clinical manifestations of nasal tumors include epistaxis, mucopurulent nasal discharge, facial deformity, and respiratory distress. These signs commonly appear once the tumor has locally infiltrated adjacent tissues [7,11]. Given the challenges of controlling tumor growth and the poor prognosis associated with advanced nasal carcinoma, treatments remain focused on local disease control. However, effective options are limited once the tumor progresses [8,12].

Radiation therapy (RT) is considered the treatment of choice for nasal tumors in dogs, regardless of the clinical stage or the histologic type of the tumor [8,10,13]. It has demonstrated superior outcomes and prolonged survival compared to other treatments, with reported median survival times ranging from 7.4 to 47.7 months depending on the protocol and tumor type [8,9,10,13]. Despite its effectiveness, local progression is common, occurring in approximately 60% of dogs treated with RT [7,9]. Acute side effects of RT, such as rhinitis, oronasal fistulas, dental pulp necrosis, and ocular complications (including keratoconjunctivitis and corneal ulcers), are frequently observed [1,7,13]. Delayed side effects and more severe complications, such as osteonecrosis and neurological toxicity, have been reported, particularly in cases that required re-irradiation, and are usually dosage-dependent [2,12,14]. Additionally, the high cost, the need for repetitive anesthesia, and the limited availability of specialized facilities make RT inaccessible for a significant number of patients [1,14]. While some dogs respond to re-irradiation following tumor recurrence, concerns about toxicity and the risk of complications often limit its use [12,13,14]. Nonetheless, RT remains the most effective treatment for controlling local disease in nasal tumors [6,10].

Surgical intervention, such as rhinotomy, is often considered a more accessible option compared to RT for the treatment of nasal tumors in dogs [15]. Surgery is highly invasive and frequently associated with significant morbidity, including acute and chronic complications such as infections and osteonecrosis [1,11]. Although ventral rhinotomy may achieve acceptable survival outcomes, the high risk of postoperative complications limits its use as a first-line treatment [16,17].

Toceranib phosphate, an antiangiogenic drug, has demonstrated effectiveness in reducing clinical signs in dogs with nasal carcinomas, offering a potential therapeutic alternative when RT is unavailable or declined by the owners [3,7,18]. Protocols using toceranib, while not curative, can be a viable palliative care option [3]. Additionally, chemotherapy protocols [19,20], and nonsteroidal anti-inflammatory drugs (NSAIDs), such as piroxicam and firocoxib, have been investigated for their potential anti-tumor effects. However, studies show limited survival benefits when used alone [9,10].

Electrochemotherapy (ECT) offers a minimally invasive alternative for treating nasal tumors, offering palliation to those with limited life expectancy or poor clinical condition. It can also serve as a cytoreductive approach to facilitate surgery without excluding other treatments [11]. Other minimally invasive options include nasal hydropulsion, which has been employed as a palliative approach, providing temporary relief from nasal obstruction in some patients [1]; cryoablation, to manage recurrent tumors [5]; and photodynamic therapy (PDT) [21], which has shown efficacy in prolonging survival in dogs with recurrent intranasal carcinomas following RT [2]. However, PDT remains limited by its high cost and the need for specialized equipment [2]. Similarly, CO_2_ laser surgery offers precise dissection and excellent hemostasis, but its applicability may be constrained by the size and location of the tumor [6].

Radiofrequency ablation (RFA) is a minimally invasive technique that uses thermal energy, generated by high-frequency alternating current, to induce coagulative necrosis in targeted tissues while preserving the surrounding structures [22,23,24]. In human medicine, RFA has been applied successfully to different tumors [25,26,27,28,29,30,31,32,33,34,35,36,37,38]. In sinonasal tumors, RFA has been shown to effectively control tumor growth [39,40]. In contrast, in veterinary medicine, while RFA and other ablation techniques have been explored as therapeutic options for other tumor types [41,42,43,44,45,46,47,48,49,50,51,52,53,54,55,56], their application in canine nasal tumors remains uninvestigated. CT-guided RFA allows precise tumor localization, minimizes the risk of damage to adjacent structures, and facilitates better control of tumor margins [57,58]. In humans, advancements in sinonasal oncologic surgery have included endoscopic transnasal RFA, which has been effective in managing sinonasal and skull base malignancies, providing optimal control of bleeding and minimal thermal damage to neural structures, such as the dura mater [59]. Furthermore, other ablative technologies, including microwave [60], low-temperature plasma radiofrequency [61,62,63,64,65], and coablation [59,66,67,68,69], have been explored in sinonasal tumor treatment, offering improved precision and reduced thermal damage. The use of High-Frequency Irreversible Electroporation (H-FIRE) has also demonstrated the potential to induce immunogenic cell death, stimulating an anti-tumor immune response and offering additional benefits beyond local ablation of the tumor [40].

The objective of this study was to evaluate the safety and feasibility of CT-guided RFA in treating nasal carcinomas, as well as to assess the volumetric reduction achieved with this technique in dogs.

## 2. Materials and Methods

Dogs with nasal masses of any size and location, whose owners had declined RT, were recruited prospectively at VetCorner (Zaragoza, Spain) from 2019 to 2024. A histologically diagnosis of nasal carcinoma, specifically the adenocarcinoma subtype, was the primary inclusion criterion. Only dogs with complete staging and histological reports reviewed and confirmed by a board-certified pathologist were included in the study. All owners signed an informed consent form before their dogs underwent the procedure described below. Previous surgeries, lesion locations, and disease stages were also documented. The clinical workup for all dogs included a complete physical examination, blood collection, chest X-rays, and a CT scan of head, neck and thorax.

**Figure 1:**
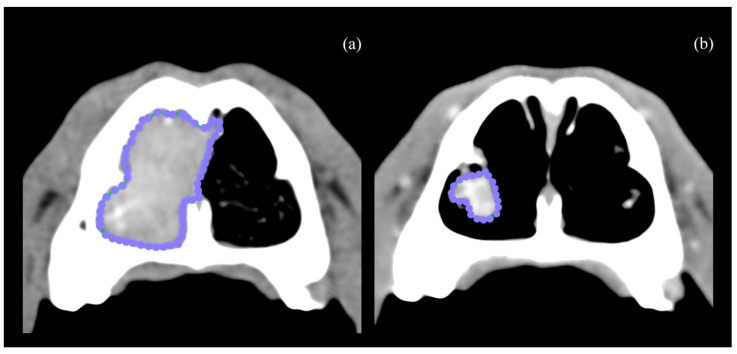
Closed polygon region of interest measured at T0 axial CT scan (**a**). CT scan from the same patient at T2 (**b**).

Computed tomography scans were performed using a 64-slice GE LightSpeed VCT (GE Healthcare, Buckinghamshire, UK). Pre- and post-contrast CT scans were acquired before the procedure (T0), and 6 weeks after the CT-guided RFA (T2). Additionally, a post-contrast CT scan was performed immediately after RFA (T1). Post-contrast series were performed following intravenous administration of 2 mL/kg of an iodinated non-ionic contrast agent (Omnipaque 300 mgI/mL solution for injection, Iohexol, GE Healthcare, Buckinghamshire, UK) using manual injection. Based on the T0 CT findings, tumors were staged according to the Adams modified system [4]. The patients were classified as D1, tumor confined to 1 nasal passage, paranasal sinus, or frontal sinus with no bony involvement; D2, tumor with bony involvement, including bilateral nasal passage involvement, but with no evidence of an orbital, subcutaneous, or submucosal mass; D3, tumor with involvement of the orbit or a subcutaneous or submucosal mass; and D4, tumor with extension into the cribriform plate. Mandibular and retropharyngeal lymph nodes were evaluated on CT images and considered enlarged if the maximum width was >10 mm or >20 mm, respectively [13]. All the volume measurements, obtained at T0, T1, and T2, were performed by a single experienced operator (A.B.-F.). A free, open-source DICOM viewer software was used to review the images (Horos, version 3.0, Horosproject.org, Nimble Co 119 LLC d/b/a Purview, Annapolis, MD, USA). The nasal tumor volumes were measured using the appropriate function in Horos and expressed in cm^3^ [8], in both pre- and post-RFA CT images (Figure 1). Nasal fluid and nasal discharge were not included in the volume measurements. The difference in contrast enhancement, measured in Hounsfield units (HU), facilitated the differentiation between the solid, contrast-enhancing mass and the non-enhancing fluid.

**Figure 2:**
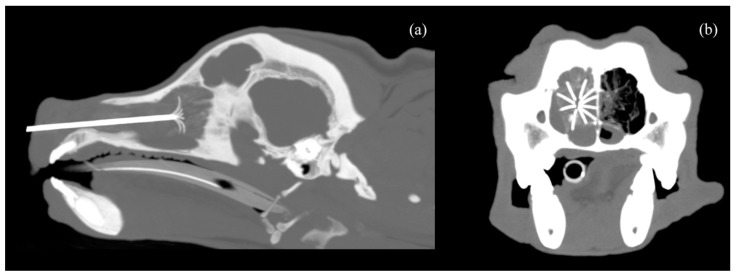
CT scan image with MIP (Maximum Intensity Projection), 7 mm thickness, in sagittal (**a**) and transverse (**b**) planes, showing the nasal carcinoma with the 2 cm LeVeen electrode fully deployed inside.

The CT-guided RFA procedure was performed under general anesthesia with the dog placed in sternal recumbence after data acquisition. All dogs were premedicated with intravenous methadone 0.2 mg/kg (Semfortan, Dechra, Northwich, UK) and diazepam 0.25 mg/kg (Ziapam, Ecuphar, Barcelona, Spain). Induction was carried out with intravenous propofol, and maintenance was performed with inhalant isoflurane. Capnography, pulse oximetry, indirect arterial blood pressure, body temperature and electrocardiogram were monitored before, during the procedure and after the thermal ablation was completed. Thermoablation was performed with an RF 3000 Radiofrequency Generator (Boston Scientific, Marlborough, MA, USA) with a LeVeen Needle Electrode (Boston Scientific, Marlborough, MA, USA), using an umbrella-like deployment configuration. The choice of electrode was based on the maximum diameter of the tumor in the cross-sectional view along the longitudinal axis of the nasal cavity. Two types of electrodes, with maximum diameters of 2 cm and 3 cm, were utilized. The 2 cm electrode was selected when the transverse diameter of the nasal cavity did not exceed 2 cm, while the 3 cm electrode was used for patients with a larger transverse diameter. Once positioned, ten atraumatic umbrella-like tines were deployed, creating a spherical ablation volume (Figure 2). 

In all cases, the electrode was initially placed at the point furthest from the nasal plane, ensuring a safety margin of at least 2 mm between the maximum diameter of the electrode and the cribriform plate, under CT guidance. At this point the monopolar radiofrequency electrode was activated, transferring electrical current from the tines to the surrounding tissue, leading to coagulation necrosis of the neoplastic tissue [23,70,71,72]. To disperse the energy produced, four adhesive electrosurgical grounding pads (3M, Saint Paul, MN, USA) were attached to the dorsal region of each hemithorax, providing a safe return path for electrosurgical currents [22]. The study protocol involved creating several ablation spheres that covered the full extent of the lesions, as the size of the tumors exceeded the diameter of the RFA needle. Once the thermal sphere was completed, the device was retracted and repositioned using the centimeter scale on the electrode. It was withdrawn from the nasal cavity by a distance equivalent to the radius used, thereby overlapping the ablation spheres. Before reactivating the radiofrequency generator for each ablation sphere, the position was verified using CT imaging. This process was repeated until the entire tumor volume was covered.

The time and power of electromagnetic energy exposure were set according to the manufacturer’s recommendations. A power algorithm starting at 10 W and gradually increasing each minute up to a maximum of 60 W was employed. Once the maximum power was reached, it was maintained until the roll-off point was achieved, characterized by an increase in impedance, as measured by the generator, which directly correlates with tissue desiccation and necrosis. At this point, the thermal ablation was deemed complete. The maximum necrosis volume was created without damaging the surrounding tissues [71]. Upon completing the procedure, the electrode was removed, and a contrast-enhanced CT scan was then performed to record the HU in the mass (T1).

The dogs were hospitalized and monitored for two hours before being discharged. After each CT-guided RFA procedure, the patients were discharged with anti-inflammatory (prednisolone 0.5 mg/kg, twice a day, orally for one week, followed by 0.25 mg/kg, twice a day, orally for five days) and antibiotic medication (amoxicillin 12.5 mg/kg, twice a day, orally for one week). Moreover, to reduce fibrinolytic activation and minimize the perioperative bleeding risk, one week before and three days after the procedure, all the dogs underwent treatment with an oral fibrinolysis inhibitor (tranexamic acid 10 mg/kg twice a day) [73,74,75]. Clinical and CT scan follow-up examinations were conducted 6 weeks after the procedure (T2).

### Statistical Analysis

The statistical description of qualitative variables was carried out with absolute and relative frequencies, and in the case of quantitative variables, were described with mean, standard deviation (SD), minimum, maximum, and quartiles. Contingency tables were calculated to assess the association between two qualitative variables, with the Likelihood Ratio test. Association between two paired variables (pre- vs. post-RFA) was assessed with the Wilcoxon test (which was selected after checking the normality of both variables with the Shapiro–Wilk test). Statistical analysis was performed with IBM SPSS 26.0 and alpha error was established in 0.05.

## 3. Results

From 2019 to 2024, 15 dogs with a histologic diagnosis of nasal carcinoma, subtype adenocarcinoma, underwent CT-guided RFA. In all cases, RT was considered prior to RFA but was declined due to economic constraints.

The included dog breeds were four (26.7%) mixed-breed, three (20%) Labrador Retrievers, three (20%) Golden Retrievers, two (13.3%) German Shepherds, one (6.7%) Pitbull, one (6.7%) Basque Shepherd, and one (6.7%) Podenco. The mean (±SD) age was 10.8 (±2.2) years, with a minimum of 6 and a maximum of 15 years. Among the dogs, 11 (73%) were male (2 intact, 9 neutered), and 4 (27%) were female (1 intact, 3 neutered).

The most frequent presenting clinical sign was nasal discharge (100%), with epistaxis observed in 13 (87%) and respiratory distress in 7 (47%) cases. In three (20%) dogs, facial deformity was present due to tumor extension into subcutaneous tissue. Several dogs received concomitant therapies: five (33%) with firocoxib, and seven (47%) with a combination of toceranib phosphate and firocoxib. NSAIDs were discontinued three days before RFA treatment and reintroduced following post-RFA corticosteroid therapy.

At T0, all patients presented with a locally destructive, attenuating, contrast-enhancing soft-tissue mass involving the turbinates on the CT scan. Evidence of nasal septum destruction was present in eight (53%) cases. Orbital involvement was observed in seven (47%) dogs, while lysis of the nasal bone accompanied by a subcutaneous mass was noted in three (20%) dogs. Extension into the cribriform plate was identified in five (33%) dogs, two (40%) of which also exhibited extension into the nasopharynx. Complete opacification of the frontal sinus, with mucous density material, was noted in 13 (87%) dogs, with total occupation in 9 (69%) cases and partial occupation in 4 (31%). No enlargement of the mandibular or retropharyngeal lymph nodes or evidence of pulmonary metastases was observed in any patient at T0.

In three (20%) dogs, nasal carcinoma was classified as stage D2; in seven (47%), as stage D3; and in five (33%), as stage D4. Three (20%) stage D4 dogs had previously undergone rhinotomy with nasal exenteration.

No significant complications were encountered during the CT-guided RFA. No significant changes were observed in capnography, pulse oximetry, indirect arterial blood pressure, body temperature, or ECG readings during the procedure, and no relevant bleeding was reported. Minor bleeding occurred during electrode insertion; however, it ceased once radiofrequency activation began. No epistaxis was reported in the perioperative period. All patients were treated as outpatients and discharged within two hours after T1.

In the post-contrast series performed at T1, an attenuation in the thermosphere areas measured in HU was documented, showing a statistically significant decrease from a mean ± SD of 98.2 ± 6.6 HU before the procedure to 60.0 ± 9.4 HU afterward (*p* = 0.001) (Table 1).

**Figure 3:**
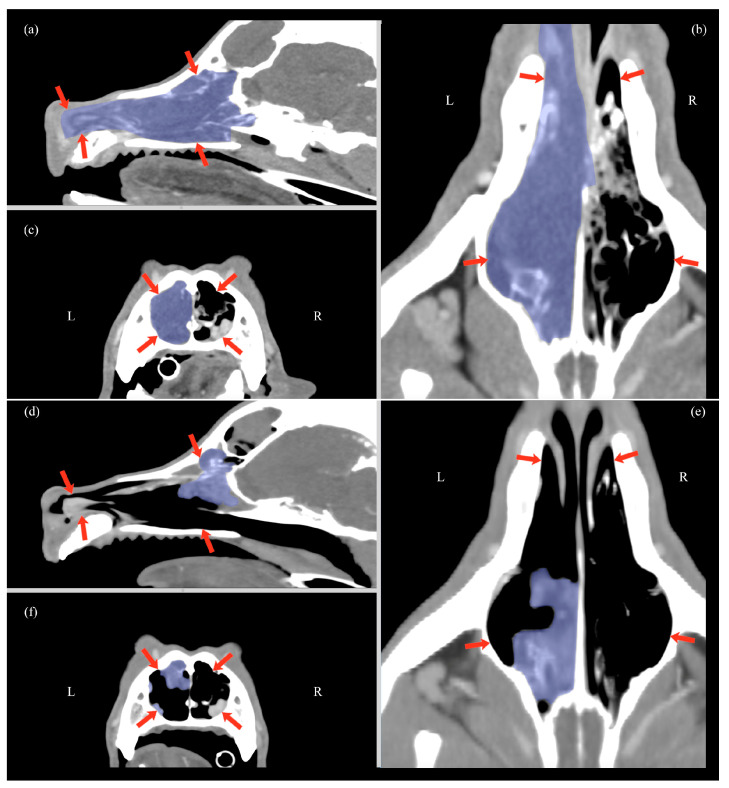
Multiplanar CT scan reconstruction at T0 (**a**–**c**) and at T2 (**d**–**f**), showing sagittal (**a**,**d**), transverse (**c**,**f**), and dorsal views (**b**,**e**). Overlain in light blue is the nasal carcinoma, a soft-tissue mass involving the turbinates. The nasal cavity is delineated by red arrows in all views.

At T2, all the patients showed resolution of initial clinical signs (nasal discharge, epistaxis, and respiratory distress) and a reduction in facial deformity. Moreover, at T2, a significant decrease in tumor volume was achieved, with an average diminution from a mean ± SD of 25.2 ± 11.1 cm^3^ to 4.4 ± 2.75 cm^3^ (*p* = 0.001), representing an 82.8% reduction (Table 1, Figure 3). The follow-up evaluation of the 13 dogs with frontal sinus opacification demonstrated improvement in all cases. None of these patients exhibited complete sinus occupation: eight (61%) had partial occupation, and one (8%) showed no residual opacification. Furthermore, three (23%) of the four dogs with partial frontal sinus involvement detected at T0 were found to have a normal sinus appearance on CT imaging at T2. No enlargement of the mandibular or retropharyngeal lymph nodes or evidence of pulmonary metastases was observed in any patient at T2.

Seven dogs (3 stage D2 and 4 stage D3) came for follow-up visits for over a year. Of these patients, three did not undergo any additional procedures within 12 months, three underwent a second CT-guided RFA at 8 and 13 months, respectively, and one underwent surgery for a growth on the external area of the nasal planum.

## 4. Discussion

The main result of this study was that CT-guided RFA in canine nasal carcinomas led to a notable improvement in clinical signs and achieved a significant reduction in tumor volume, highlighting its potential as a viable treatment option for nasal tumors in dogs.

Canine nasal carcinomas are locally invasive malignancies characterized by a low rate of metastasis [9,76,77,78]. Cytoreductive local control is currently the cornerstone in the management of these patients. Radiation therapy is considered the treatment of choice; however, it has significant limitations, including the high cost, the need for repeated anesthesia, and limited availability [7,8,12,13,14,40,78]. Furthermore, this modality has been associated with the development of acute and delayed adverse effects such as rhinitis, oronasal fistula formation, pulp necrosis, and varying degrees of ocular, cutaneous, and neurological toxicity [7,8,12,13,14,40,78]. In addition, repeated radiation exposure can increase the risk of serious complications, including osteonecrosis and ocular damage [12,14,77]. Surgical exenteration (via rhinotomy) is also a viable option, though it is equally expensive and often associated with a higher incidence of postoperative complications, such as infections and osteonecrosis. Consequently, the high morbidity rate limits its use [1]. Ongoing research on this disease aims to identify therapeutic approaches that can enhance outcomes without compromising the safety profile of the treatments [39].

Radiofrequency ablation is a minimally invasive procedure widely used in human medicine to treat various types of tumors, including multiple sinonasal neoplasms [22,23,24]. This technique offers the advantage of reducing tumor size while minimizing damage to surrounding tissues [39,57,58]. In this study, RFA achieved a significant (82.8%) tumor volume reduction. A retrospective study indicates that a nasal exenteration may be indicated if, following a course of RT, tumor volume regression assessed via CT is less than 80% [16]. In our study, the results demonstrate a tumor volume reduction exceeding this 80% threshold. For instance, Morgan et al. (2018) reported a 67.1% decrease in tumor volume for canine nasal carcinomas [8], while Bommarito et al. (2011) demonstrated a median volume regression of 85.6% after the first course of RT [12]. These findings suggest that RFA offers a comparable initial degree of tumor control to RT. The improvement in mucous material occupancy within the frontal sinus further highlights the significant degree of tumor volume reduction. It is likely that the clearance of the sinus also contributed substantially to the clinical improvement observed in the patients.

Although complete necrosis typically occurs within 7 to 10 days following the procedure [22], it is established that maximum tumor volume reduction occurs between 4 and 8 weeks after RFA [22,79]. Therefore, the follow-up visit, and CT scan were scheduled 6 weeks after the procedure. The decrease in HU immediately after the RFA (T1) demonstrates the effect of radiofrequency on contrast uptake within the thermosphere. This tissue necrosis, accompanied by vascular destruction, is the primary factor responsible for cellular death. Post-contrast CT is particularly useful for identifying large blood vessels that could dissipate heat and reduce the effectiveness of the thermosphere. Additionally, the decrease in HU serves to evaluate whether the planned volume in the ballistic targeting of the mass has been successfully achieved. In areas near large vessels, greater overlapping of spheres can be planned to enhance the likelihood of achieving necrosis. However, RFA is a local treatment with inherent limitations. Tumor regions that are difficult to access with the electrode may persist and continue to progress. Moreover, the presence of large vessels proximal to the thermosphere can diminish the efficacy of the treatment due to the “heat sink effect” [22]. This contrasts with RT which exerts a broader impact on the entire targeted area [8,13].

Radiofrequency ablation has demonstrated a very high safety profile, with no serious adverse effects observed. The nasal cavity is highly vascularized and RFA, although minimally invasive, still causes some trauma. Minimal bleeding should be expected as a common side effect, the same way it occurs during highly invasive procedures associated with a higher risk of serious complications such as surgery [6,16,76]. However, as with ECT, RFA induces a phenomenon known as the vascular lock, which helps reduce bleeding both during and after the procedure [11,80]. This occurs due to the thermal coagulation of surrounding blood vessels as the heat generated by the RFA destroys the tissue. The elevated temperature effectively seals the vessels, resulting in immediate coagulation and minimizing the risk of hemorrhage. The vascular lock is one of the advantages of RFA compared to other procedures [58,59,67,68]. As described in human medicine, RFA provides optimal bleeding control and causes minimal thermal damage to adjacent tissues [39].

Local invasiveness and recurrence are among the main characteristics of nasal carcinomas. In this study, five patients who previously underwent nasal exenteration were included. Although the number of cases is limited, no significant difference was observed in tissue reduction response in patients treated surgically before RFA. Similarly, RFA could be considered for patients with recurrence after RT, potentially avoiding or delaying reirradiation. Additionally, some studies recommend surgical cytoreduction following RT if a macroscopically visible residual tumor is present [4,13,78,81], with the purpose of delaying recurrence [13,81]. Radiofrequency ablation could be considered as an alternative for patients for which a surgical approach or RT is not feasible.

An advantage of RFA is its potential use as a component of multimodal approaches. In this study, seven dogs were receiving toceranib when RFA was performed. It is not possible to ascertain whether there is a correlation between the response to radiofrequency and concomitant treatment with toceranib phosphate. However, previous studies in human medicine have demonstrated the efficacy of RFA and other thermoablative therapies when applied in conjunction with chemotherapeutic protocols, immunotherapy, or surgical approaches [82,83,84,85,86].

Another aspect that should be investigated in the future is the potential immunomodulatory effect that RFA might generate in nasal carcinomas. It has been demonstrated that different types of nasal tumors elicit varying immune responses [87]. After ablation, tumor antigens instantly become available to leukocytes, and the procedure creates an inflammatory environment that may help stimulate both innate and adaptive anti-tumor immunity [88]. Combining tumor debulking by RFA with immune-stimulatory approaches that enhance antigen presentation and promote anti-tumor T cell reactivity is a promising strategy to prevent local recurrences and induce long-term systemic protection against residual disease [40,88,89,90]. A recent study on canine lung tumors demonstrated that irreversible electroporation induces local necrosis and significant apoptosis, marked by increased cleaved caspase-3. Post-treatment, the tumor microenvironment showed immunomodulatory changes, including altered macrophage infiltration and significant gene expression changes related to inflammation and immune response, such as IDO1, IL-6, TNF, CD209, and FOXP3 [40]. 

The effectiveness of RFA in non-carcinomatous nasal tumors remains an area for future exploration. Tumor sensitivity to RT is influenced by the α/β ratio, with carcinomas generally showing a higher ratio and better response compared to sarcomas, which tend to have a lower α/β ratio. Sarcomas, due to their heterogeneity and complex vascularization, respond less effectively to RFA, particularly in primary soft tissue sarcomas [8,91,92].

This study presents several limitations. Firstly, only patients with a histopathological diagnosis of nasal adenocarcinoma were recruited. While it is the most common, other carcinoma subtypes, such as squamous cell carcinoma or undifferentiated carcinoma, were excluded. The decision to exclude nasal tumors other than adenocarcinomas was based on two considerations: first, the aim to maintain a homogeneous population of clinical cases with a consistent histological tumor type; and second, the fact that adenocarcinomas are the most prevalent nasal neoplasms in dogs [3,13,77,78]. Taking into consideration that these tumor types have different survival rates and behavior [78], our results cannot be generalized to other carcinomas or other types of nasal tumors such as fibrosarcoma, chondrosarcoma, osteosarcoma, or lymphomas.

A second limitation was that we did not know the number of dogs that had undergone cytological examination of the mandibular lymph nodes prior to the study. At T0 and T2, lymph nodal and pulmonary metastasis were ruled out based on the radiological criterion of lymph node size on the CT scan.

Reporting a disease-free interval or a median survival time following the CT-guided RFA falls outside the scope of the present study. Although the initial tumor volume reduction results are very promising, larger studies with mid-term and long-term follow-ups are needed to better define the benefits of RFA in nasal adenocarcinomas in dogs.

## 5. Conclusions

Radiofrequency ablation in nasal adenocarcinomas appears to be a safe and feasible procedure, achieving significant tumor volume reduction and improvement in clinical signs without significant adverse events. This procedure could be considered as an alternative to RT or surgery when these strategies are discarded, or as a cytoreductive strategy in cases of recurrence or partial response.

## Figures and Tables

**Table 1:** Hounsfield units at T0 and T1; percentage of attenuation reduction; and tumor volume at T0 and T2.

Variable	Time	Mean	SD	min	Q1	Q2	Q3	Max	*p*-Value ^w^
HU	T0	98.2	6.6	87	95	98	101.5	110	0.001
	T1	60.9	9.4	50	52.5	61	67.5	78	
Attenuation reduction (%)		37.6	11.1	11.4	32.4	38	46.1	51.8	
Volume (cm^3^)	T0	25.2	11.1	12.3	17.0	19.5	35.7	43.8	0.001
	T2	4.4	2.7	2.2	2.6	3.2	4.8	11.2	
Volume reduction (%)		82.8	4.5	74.4	81.5	83.1	84.7	9.1	

HU, Hounsfield units; T0, CT scan before RFA; T1, CT scan immediately after RFA; T2, CT scan 6 weeks after RFA; ^w^, statistical significance according to Wilcoxon test.

## Data Availability

The original data presented in the study are openly available in FigShare at https://doi.org/10.6084/m9.figshare.27902472.

## References

[1] Bienes T., Robin E., Le Boedec K. (2019). Hydropulsion as Palliative, Long-Term, Last-Resort Treatment of Nasal Carcinoma in a Dog and a Cat. J. Am. Anim. Hosp. Assoc..

[2] Ishigaki K., Nariai K., Izumi M., Teshima K., Seki M., Edamura K., Takahashi T., Asano K. (2018). Endoscopic photodynamic therapy using talaporfin sodium for recurrent intranasal carcinomas after radiotherapy in three dogs. J. Small Anim. Pract..

[3] Merino-Gutierrez V., Borrego J.F., Puig J., Hernández A., Clemente-Vicario F. (2021). Treatment of advanced-stage canine nasal carcinomas with toceranib phosphate: 23 cases (2015–2020). J. Small Anim. Pract..

[4] Adams W.M., Kleiter M.M., Thrall D.E., Klauer J.M., Forrest L.J., La Due T.A., Havighurst T.C. (2009). Prognostic significance of tumor histology and computed tomographic staging for radiation treatment response of canine nasal tumors. Vet. Radiol. Ultrasound.

[5] Murphy S.M., Lawrence J.A., Schmiedt C.W., Davis K.W., Lee F.T., Forrest L.J., Bjorling D.E. (2011). Image-guided transnasal cryoablation of a recurrent nasal adenocarcinoma in a dog. J. Small Anim. Pract..

[6] Carreira L.M., Azevedo P. (2024). Advantages of the co(2) laser use in the rare condition of nasal mucosa squamous cell carcinoma surgery in dogs-a clinical prospective study. Lasers Med. Sci..

[7] Ehling T.J., Klein M.K., Smith L., Prescott D., Haney S., Looper J., LaDue T., Brawner W., Fidel J., Shiomitsu K. (2022). A prospective, multi-centre, Veterinary Radiation Therapy Oncology Group study reveals potential efficacy of toceranib phosphate (Palladia) as a primary or adjuvant agent in the treatment of canine nasal carcinoma. Vet. Comp. Oncol..

[8] Morgan M.J., Lurie D.M., Villamil A.J. (2018). Evaluation of tumor volume reduction of nasal carcinomas versus sarcomas in dogs treated with definitive fractionated megavoltage radiation: 15 cases (2010-2016). BMC Res. Notes.

[9] Rassnick K.M., Goldkamp C.E., Erb H.N., Scrivani P.V., Njaa B.L., Gieger T.L., Turek M.M., McNiel E.A., Proulx D.R., Chun R. (2006). Evaluation of factors associated with survival in dogs with untreated nasal carcinomas: 139 cases (1993–2003). J. Am. Vet. Med. Assoc..

[10] Cancedda S., Sabattini S., Bettini G., Leone V.F., Laganga P., Rossi F., Terragni R., Gnudi G., Vignoli M. (2015). Combination of radiation therapy and firocoxib for the treatment of canine nasal carcinoma. Vet. Radiol. Ultrasound.

[11] Maglietti F., Tellado M., Olaiz N., Michinski S., Marshall G. (2017). Minimally Invasive Electrochemotherapy Procedure for Treating Nasal Duct Tumors in Dogs using a Single Needle Electrode. Radiol. Oncol..

[12] Bommarito D.A., Kent M.S., Selting K.A., Henry C.J., Lattimer J.C. (2011). Reirradiation of recurrent canine nasal tumors. Vet. Radiol. Ultrasound.

[13] Mayer M.N., DeWalt J.O., Sidhu N., Mauldin G.N., Waldner C.L. (2019). Outcomes and adverse effects associated with stereotactic body radiation therapy in dogs with nasal tumors: 28 cases (2011–2016). J. Am. Vet. Med. Assoc..

[14] Gieger T.L., Haney S.M., Nolan M.W. (2022). Re-irradiation of canine non-lymphomatous nasal tumours using stereotactic radiation therapy (10 Gy x 3) for both courses: Assessment of outcome and toxicity in 11 dogs. Vet. Comp. Oncol..

[15] Dickerson V.M., Grimes J.A., Vetter C.A., Colopy S.A., Duval J.M., Northrup N.C., Schmiedt C.W. (2019). Outcome following cosmetic rostral nasal reconstruction after planectomy in 26 dogs. Vet. Surg..

[16] Adams W.M., Bjorling D.E., McAnulty J.E., Green E.M., Forrest L.J., Vail D.M. (2005). Outcome of accelerated radiotherapy alone or accelerated radiotherapy followed by exenteration of the nasal cavity in dogs with intranasal neoplasia: 53 cases (1990–2002). J. Am. Vet. Med. Assoc..

[17] Laing E.J., Binnington A.G. (1988). Surgical therapy of canine nasal tumors: A retrospective study (1982–1986). Can. Vet. J..

[18] Pauly L.A.M., Junginger J., Oechtering G.U., Hewicker-Trautwein M., Rösch S. (2024). Expression of vascular endothelial growth factor receptor-2, epidermal growth factor receptor, cyclooxygenase-2, survivin, E-cadherin and Ki-67 in canine nasal carcinomas and sarcomas—A pilot study. Front. Vet. Sci..

[19] Giuliano A., Almendros A. (2022). Retrospective Evaluation of a Combination of Carboplatin and Bleomycin for the Treatment of Canine Carcinomas. Animals.

[20] Woodruff M.J., Heading K.L., Bennett P. (2019). Canine intranasal tumours treated with alternating carboplatin and doxorubin in conjunction with oral piroxicam: 29 cases. Vet. Comp. Oncol..

[21] Dobson J., de Queiroz G.F., Golding J.P. (2018). Photodynamic therapy and diagnosis: Principles and comparative aspects. Vet. J..

[22] Ellis L.M., Curley C.A., Tanabe K.K. (2004). Radiofrequency Ablation: Current Indications, Techniques and Outcomes.

[23] Singh S., Melnik R. (2020). Thermal ablation of biological tissues in disease treatment: A review of computational models and future directions. Electromagn. Biol. Med..

[24] Vogt F.M., Antoch G., Veit P., Freudenberg L.S., Blechschmid N., Diersch O., Bockisch A., Barkhausen J., Kuehl H. (2007). Morphologic and functional changes in nontumorous liver tissue after radiofrequency ablation in an in vivo model: Comparison of 18F-FDG PET/CT, MRI, ultrasound, and CT. J. Nucl. Med..

[25] Alyusuf E.Y., Ekhzaimy A.A., Rivera J.A. (2021). Radiofrequency Ablation as a Primary Therapy for Benign Functioning Insulinoma. AACE Clin. Case Rep..

[26] Bai X.M., Cui M., Yang W., Wang H., Wang S., Zhang Z.Y., Wu W., Chen M.H., Yan K., Goldberg S.N. (2021). The 10-year Survival Analysis of Radiofrequency Ablation for Solitary Hepatocellular Carcinoma 5 cm or Smaller: Primary versus Recurrent HCC. Radiology.

[27] Dai Y., Covarrubias D., Uppot R., Arellano R.S. (2017). Image-Guided Percutaneous Radiofrequency Ablation of Central Renal Cell Carcinoma: Assessment of Clinical Efficacy and Safety in 31 Tumors. J. Vasc. Interv. Radiol..

[28] Galletti B., Gazia F., Galletti C., Freni F., Galletti C., Bruno R., Sireci F., Galletti F. (2021). Radiofrequency VS Cold Surgery to Treat Oral Papillomatous Lesions. Iran. J. Otorhinolaryngol..

[29] Hasegawa T., Kuroda H., Sakakura N., Sato Y., Chatani S., Murata S., Yamaura H., Nakada T., Oya Y., Inaba Y. (2021). Novel strategy to treat lung metastases: Hybrid therapy involving surgery and radiofrequency ablation. Thorac. Cancer.

[30] Inoue T., Yoneda M. (2021). Updated evidence on the clinical impact of endoscopic radiofrequency ablation in the treatment of malignant biliary obstruction. Dig. Endosc..

[31] Koo J.S., Chung S.H. (2021). The Efficacy of Radiofrequency Ablation for Bone Tumors Unsuitable for Radical Excision. Clin. Orthop. Surg..

[32] Nunes T.F., Szejnfeld D., Xavier A.C., Goldman S.M. (2013). Percutaneous ablation of functioning adenoma in a patient with a single adrenal gland. BMJ Case Rep..

[33] Nunes T.F., Szejnfeld D., Xavier A.C., Kater C.E., Freire F., Ribeiro C.A., Goldman S.M. (2013). Percutaneous ablation of functioning adrenal adenoma: A report on 11 cases and a review of the literature. Abdom. Imaging.

[34] Qu C., Li X.Q., Li C., Xia F., Feng K., Ma K. (2021). The Short-Term Efficacy of Novel No-Touch Combined Directional Perfusion Radiofrequency Ablation in the Treatment of Small Hepatocellular Carcinoma with Cirrhosis. J. Investig. Surg..

[35] Rimbaș M., Rizzatti G., Larghi A. (2021). EUS-guided ablation of pancreatic neoplasms. Minerva Gastroenterol..

[36] Shen X., Chen T., Yang B., Liu N., Qian X., Xia B., Feng D., Chen S. (2021). Magnetic resonance imaging-guided microwave ablation for lung tumor: A case report. Quant. Imaging Med. Surg..

[37] Shibamoto K., Mimura H., Fukuhara Y., Nishino K., Kawamoto H., Kato K. (2021). Feasibility, safety, and efficacy of artificial carbon dioxide pneumothorax for computed tomography fluoroscopy-guided percutaneous radiofrequency ablation of hepatocellular carcinoma. Jpn. J. Radiol..

[38] Wu M.H., Chen K.Y., Chen A., Chen C.N. (2021). Differences in the ultrasonographic appearance of thyroid nodules after radiofrequency ablation. Clin. Endocrinol..

[39] Ferrari M., Orlandi E., Bossi P. (2021). Sinonasal cancers treatments: State of the art. Curr. Opin. Oncol..

[40] Hay A.N., Aycock K.N., Lorenzo M.F., David K., Coutermarsh-Ott S., Salameh Z., Campelo S.N., Arroyo J.P., Ciepluch B., Daniel G. (2024). Investigation of High Frequency Irreversible Electroporation for Canine Spontaneous Primary Lung Tumor Ablation. Biomedicines.

[41] Zhong C.H., Fan M.Y., Xu H., Jin R.G., Chen Y., Chen X.B., Tang C.L., Su Z.Q., Li S.Y. (2021). Feasibility and Safety of Radiofrequency Ablation Guided by Bronchoscopic Transparenchymal Nodule Access in Canines. Respiration.

[42] Yang T., Case J.B., Boston S., Dark M.J., Toskich B. (2017). Microwave ablation for treatment of hepatic neoplasia in five dogs. J. Am. Vet. Med. Assoc..

[43] Solari F.P., Case J.B., Vilaplana Grosso F.R., Bertran J., Fox-Alvarez S., Cabrera R. (2024). Laparoscopic ultrasound-guided microwave ablation of hepatocellular carcinoma in a dog. Vet. Surg..

[44] Partridge B.R., O’Brien T.J., Lorenzo M.F., Coutermarsh-Ott S.L., Barry S.L., Stadler K., Muro N., Meyerhoeffer M., Allen I.C., Davalos R.V. (2020). High-Frequency Irreversible Electroporation for Treatment of Primary Liver Cancer: A Proof-of-Principle Study in Canine Hepatocellular Carcinoma. J. Vasc. Interv. Radiol..

[45] Mazzaccari K., Boston S.E., Toskich B.B., Bowles K., Case J.B. (2017). Video-assisted microwave ablation for the treatment of a metastatic lung lesion in a dog with appendicular osteosarcoma and hypertrophic osteopathy. Vet. Surg..

[46] Locatelli A., Treggiari E., Innocenti M., Romanelli G. (2022). Percutaneous ultrasound-guided microwave ablation for treatment of hepatocellular carcinomas in dogs: Four cases (2019-2020). J. Small Anim. Pract..

[47] Liu R., Duan S., Cao H., Cao G., Chang Z., Zhang Y., Li Y., Wu Y., Liu L., Zhang L. (2020). A pilot study of the shapes of ablation lesions in the canine prostate by laser, radiofrequency and microwave and their clinical significance. PLoS ONE.

[48] Jia L., Bin H., Bing H., Jin H. (2021). CEUS examination of the outcome of radiofrequency ablation of canine prostate lesions. Minim. Invasive Ther. Allied Technol..

[49] Hung A.J., Ma Y., Zehnder P., Nakamoto M., Gill I.S., Ukimura O. (2012). Percutaneous radiofrequency ablation of virtual tumours in canine kidney using Global Positioning System-like technology. BJU Int..

[50] Hu B., Hu B., Chen L., Li J., Huang J. (2010). Contrast-enhanced ultrasonography evaluation of radiofrequency ablation of the prostate: A canine model. J. Endourol..

[51] Gomez Ochoa P., Alferez M.D., de Blas I., Fernendes T., Sanchez Salguero X., Balana B., Melendez Lazo A., Barbero Fernandez A., Caivano D., Corda F. (2021). Ultrasound-Guided Radiofrequency Ablation of Chemodectomas in Five Dogs. Animals.

[52] Dornbusch J.A., Wavreille V.A., Dent B., Fuerst J.A., Green E.M., Selmic L.E. (2020). Percutaneous microwave ablation of solitary presumptive pulmonary metastases in two dogs with appendicular osteosarcoma. Vet. Surg..

[53] Culp W.T.N., Johnson E.G., Palm C.A., Burton J.H., Rebhun R.B., Rodriguez C.O., Kent M.S., Glaiberman C.B. (2021). Use of percutaneous microwave ablation in the treatment of retroperitoneal neoplasia in three dogs. J. Am. Vet. Med. Assoc..

[54] Chen W., Tang X., Yang X., Weng C., Yang K., Wen J., Liu H., Wu Y. (2017). Effects and Mechanisms of Radiofrequency Ablation of Renal Sympathetic Nerve on Anti-Hypertension in Canine. Arq. Bras. Cardiol..

[55] Carroll J., Coutermarsh-Ott S., Klahn S.L., Tuohy J., Barry S.L., Allen I.C., Hay A.N., Ruth J., Dervisis N. (2022). High intensity focused ultrasound for the treatment of solid tumors: A pilot study in canine cancer patients. Int. J. Hyperth..

[56] Alférez M.D., Corda A., de Blas I., Gago L., Fernandes T., Rodríguez-Piza I., Balañá B., Corda F., Gómez Ochoa P. (2024). Percutaneous Ultrasound-Guided Radiofrequency Ablation as a Therapeutic Approach for the Management of Insulinomas and Associated Metastases in Dogs. Animals.

[57] Du W. (2013). Effect analysis of nasal inverted papilloma in nasal cavity and paranasal sinus by radiofrequency ablation under nasal endoscopy. Lin Chuang Er Bi Yan Hou Tou Jing Wai Ke Za Zhi.

[58] Liang J.P., Li D.Y., Liu B. (2000). Radiofrequency treatment of hemangioma of nasal cavity under nasal endoscopy. Lin Chuang Er Bi Yan Hou Ke Za Zhi.

[59] Kostrzewa J.P., Sunde J., Riley K.O., Woodworth B.A. (2010). Radiofrequency coblation decreases blood loss during endoscopic sinonasal and skull base tumor removal. ORL J. Otorhinolaryngol. Relat. Spec..

[60] Lou Z.C. (2020). Microwave Ablation for the Removal of Benign Lesion of Nasal Cavity: “How I Do It”. Am. J. Rhinol. Allergy.

[61] Long X., Li Z., Liu Y., Zhen H. (2024). Clinical Application of Low-Temperature Plasma Radiofrequency in the Treatment of Hemangioma in Nasal Cavity, Pharynx and Larynx. Ear Nose Throat J..

[62] She C.P., Zhang Q.F., Song W., Zhang X.R., Cheng C.J., Pan T. (2010). Endoscopic surgery using the low-temperature plasma radiofrequency for nasal hemangioma. Zhonghua Er Bi Yan Hou Tou Jing Wai Ke Za Zhi.

[63] Zhang D., Xiao L., Tian H. (2014). Endoscopic pleomorphic adenoma of nasal septum resection assisted by low-temperature plasm radiofrequency: A case report. Lin Chuang Er Bi Yan Hou Tou Jing Wai Ke Za Zhi.

[64] Zhang Q., She C., Song W., Cui S. (2014). Nasal mucosa recovery after endoscopic surgery using the plasma radiofrequency ablation at low temperature for treatment of nasal inverted papilloma. Lin Chuang Er Bi Yan Hou Tou Jing Wai Ke Za Zhi.

[65] Zhong Q.Y., Sun Q., Liu Z.H. (2021). Endoscopic Low-Temperature Plasma Radiofrequency Ablation for Primary Thyroid-Like Low-Grade Nasopharyngeal Papillary Adenocarcinoma. Ear Nose Throat J..

[66] Cannon D.E., Poetker D.M., Loehrl T.A., Chun R.H. (2013). Use of coblation in resection of juvenile nasopharyngeal angiofibroma. Ann. Otol. Rhinol. Laryngol..

[67] Ruiz J.W., Saint-Victor S., Tessema B., Eloy J.A., Anstead A. (2012). Coblation assisted endoscopic juvenile nasopharyngeal angiofibroma resection. Int. J. Pediatr. Otorhinolaryngol..

[68] Syed M.I., Mennie J., Williams A.T. (2012). Early experience of radio frequency coblation in the management of intranasal and sinus tumors. Laryngoscope.

[69] Zhang L., Shi H., Li D., Ye H., Zhang W., Yin S. (2020). Radiofrequency Coblation-Assisted Resection of Skull Base Neoplasms Using an Endoscopic Endonasal Approach. ORL J. Otorhinolaryngol. Relat. Spec..

[70] Goldberg S.N., Gazelle G.S., Compton C.C., Mueller P.R., Tanabe K.K. (2000). Treatment of intrahepatic malignancy with radiofrequency ablation: Radiologic-pathologic correlation. Cancer.

[71] Jiang B., Zhao K., Yan K., Wang S., Meng Y., Liu B., Wu H., Wang H. (2021). Percutaneous radiofrequency ablation near large vessels in beagle livers: The impact of time and distance on the ablation zone. Int. J. Hyperth..

[72] Wood B.J., Abraham J., Hvizda J.L., Alexander H.R., Fojo T. (2003). Radiofrequency ablation of adrenal tumors and adrenocortical carcinoma metastases. Cancer.

[73] Faraoni D., Willems A., Melot C., De Hert S., Van der Linden P. (2012). Efficacy of tranexamic acid in paediatric cardiac surgery: A systematic review and meta-analysis. Eur. J. Cardiothorac. Surg..

[74] Fletcher D.J., Blackstock K.J., Epstein K., Brainard B.M. (2014). Evaluation of tranexamic acid and epsilon-aminocaproic acid concentrations required to inhibit fibrinolysis in plasma of dogs and humans. Am. J. Vet. Res..

[75] Marin L.M., Iazbik M.C., Zaldivar-Lopez S., Guillaumin J., McLoughlin M.A., Couto C.G. (2012). Epsilon aminocaproic acid for the prevention of delayed postoperative bleeding in retired racing greyhounds undergoing gonadectomy. Vet. Surg..

[76] Henry C.J., Brewer W.G., Tyler J.W., Brawner W.R., Henderson R.A., Hankes G.H., Royer N. (1998). Survival in dogs with nasal adenocarcinoma: 64 cases (1981-1995). J. Vet. Intern. Med..

[77] Malinowski C. (2006). Canine and feline nasal neoplasia. Clin. Tech. Small Anim. Pract..

[78] Mizuno R., Mori T. (2024). Prognostic factors and survival following radiation therapy for canine nasal tumors: A single-institution retrospective study of 166 cases. Open Vet. J..

[79] Nemcek A.A. (2006). Complications of radiofrequency ablation of neoplasms. Semin. Interv. Radiol..

[80] Mir L.M., Morsli N., Garbay J.R., Billard V., Robert C., Marty M. (2003). Electrochemotherapy: A new treatment of solid tumors. J. Exp. Clin. Cancer Res..

[81] Hunley D.W., Mauldin G.N., Shiomitsu K., Mauldin G.E. (2010). Clinical outcome in dogs with nasal tumors treated with intensity-modulated radiation therapy. Can. Vet. J..

[82] Zhang Y., Sun Y., Chen J., Wang X., Wu K., Huang Z. (2017). Microwave ablation combined with neoadjuvant chemotherapy for the treatment of breast cancer: A randomized controlled trial. Oncotarget.

[83] Song M.J., Bae S.H., Lee J.S., Lee S.W., Song D.S., You C.R., Choi J.Y., Yoon S.K. (2019). Radiofrequency ablation plus drug-eluting beads transcatheter arterial chemoembolization for the treatment of single large hepatocellular carcinoma. Liver Int..

[84] Liu H., Wang Z.G., Fu S.Y., Li A.J., Pan Z.Y., Zhou W.P., Lau W.Y., Wu M.C. (2016). Combination of radiofrequency ablation with transarterial chemoembolization for hepatocellular carcinoma: A multicentre randomized trial. J. Hepatol..

[85] Kim J.W., Kim J.H., Won H.J., Shin Y.M., Yoon H.K., Sung K.B., Kim P.N. (2014). Percutaneous radiofrequency ablation combined with transcatheter arterial chemoembolization and ethanol injection for hepatocellular carcinoma 3–5 cm in diameter. Eur. Radiol..

[86] Greten T.F., Mauda-Havakuk M., Heinrich B., Korangy F., Wood B.J. (2020). Combination radiofrequency ablation and immunotherapy in unresectable hepatocellular carcinoma: A randomized phase II trial. J. Clin. Oncol..

[87] Vanherberghen M., Day M.J., Delvaux F., Gabriel A., Clercx C., Peeters D. (2009). An immunohistochemical study of the inflammatory infiltrate associated with nasal carcinoma in dogs and cats. J. Comp. Pathol..

[88] Nierkens S., den Brok M.H., Ruers T.J., Adema G.J., Keisari Y. (2013). Radiofrequency Ablation in Cancer Therapy: Tuning in to in situ Tumor Vaccines. Tumor Ablation: Effects on Systemic and Local Anti-Tumor Immunity and on Other Tumor-Microenvironment Interactions.

[89] Gameiro S.R., Higgins J.P., Dreher M.R., Woods D.L., Reddy G., Wood B.J., Guha C., Hodge J.W. (2013). Combination therapy with local radiofrequency ablation and systemic vaccine enhances antitumor immunity and mediates local and distal tumor regression. PLoS ONE.

[90] Slovak R., Ludwig J.M., Gettinger S.N., Herbst R.S., Kim H.S. (2017). Immuno-thermal ablations—Boosting the anticancer immune response. J. Immunother. Cancer.

[91] Palussière J., Italiano A., Descat E., Ferron S., Cornélis F., Avril A., Brouste V., Bui B.N. (2011). Sarcoma Lung Metastases Treated with Percutaneous Radiofrequency Ablation: Results from 29 Patients. Ann. Surg. Oncol..

[92] Parvinian A., Thompson S.M., Schmitz J.J., Welch B.T., Hibbert R., Adamo D.A., Kurup A.N. (2024). Update on Percutaneous Ablation for Sarcoma. Curr. Oncol. Rep..

